# Understanding competency development of the management workforce in veterinary clinical practice: A scoping review

**DOI:** 10.1002/vro2.70011

**Published:** 2025-05-19

**Authors:** Taleta Hompas, Zhanming Liang

**Affiliations:** ^1^ College of Business, Law and Governance, James Cook University Townsville Queensland Australia

**Keywords:** capacity building, leadership, management competency, veterinary

## Abstract

**Background:**

The historical challenges facing veterinary care have been exacerbated by the surge in pet ownership and demand for services following the COVID‐19 pandemic. Managers in veterinary services are essential in navigating these challenges while ensuring the provision of quality animal care. Evidence from human healthcare highlights the importance of developing and supporting managers to thrive in their management roles. However, there is currently no established management competency framework to guide development of managers working in veterinary care.

**Methods:**

A two‐step approach was used to explore the existing efforts to develop a competent veterinary management workforce. A scoping review of Web of Science, PubMed and Scopus databases was conducted using the Arksey and O'Malley framework. This review aimed to identify both the key management challenges in veterinary services and specific competencies required by veterinary managers. A supplementary web‐based search of professional institutions and postgraduate programmes relevant to veterinary leadership and management was also completed.

**Results:**

Fourteen articles met the inclusion criteria with only five of them published after 2011. The literature identified key challenges in managing veterinary services and revealed a limited recognition of skills and competencies for veterinary managers, especially when compared with the human healthcare sector. Several professional institutions and educational programmes supporting veterinary leaders were identified.

**Conclusion:**

Findings confirm that current approaches to management competency identification and development in veterinary care are insufficient. A validated, comprehensive framework to build management capability in veterinary care is urgently needed to support managers in fulfilling their roles and to promote sustainable veterinary service delivery.

## INTRODUCTION

Evidence has emerged over the past two decades that indicates the sustainability of veterinary clinical care services to provide high‐quality animal care has been threatened by high levels of attrition among veterinary professionals, attributed by high rates of burnout, stress and negative mental health outcomes.^1^, ^2^, ^3^, ^4^, ^5^ Historical factors such as governmental underinvestment, low remuneration and significant education debt make veterinary care unattractive to new graduates, which has further exacerbated workforce shortages.^2^, ^6^, ^7^ On the other hand, the unprecedented surge in pet ownership and demand for veterinary services following the COVID‐19 pandemic has further reduced veterinary care services’ capacity to meet the increased demand for animal care.^8^ Veterinary practice has been pressured to improve effectiveness and efficiency which may rely on improved organisation structures and processes, a skilled veterinary workforce and competent managers who are responsible for the strategic development and day‐to‐day operation of the veterinary care services.

However, a low level of management skills characterises the veterinary profession and many veterinarians who are clinically competent may lack some of the crucial non‐clinical skills, knowledge, aptitudes and attitudes (SKAAs) that are correlated with economic success.^3^, ^4^ This gap was acknowledged in graduate veterinarians, who did not possess the SKAAs consistent with levels desired by their employers more than two decades ago.^2^ These management skills are necessary at every level of the veterinary profession to enable veterinary professionals to effectively manage the new technologies, expanding scientific knowledge and changing societal expectations and therefore retaining their position as leaders within the local community and society.

Management positions within veterinary care organisations have traditionally been filled by clinical veterinary staff who have been promoted to management positions based on their clinical competence but do not possess management qualification(s) or substantial management training.^3^, ^4^ Unclear management development pathways as well as a lack of preparedness of veterinary care professionals taking on management roles commonly result in stressful transitions to leadership positions and an increased inability to manage work and life balance.^5^, ^7^ A current crisis in all sectors (public, private, corporate) of veterinary practice exists because of poor work‒life integration, professional underachievement and limited non‐technical competencies. In 2018, one study detailed that this experience was true for veterinarians placed in management positions because of the inappropriate training and support in transition to these roles and was exacerbated by factors such as the challenges of corporate practice ownership, the growing shortage of veterinary surgeons and a turbulent macroenvironment.^5^ Increased demand for managers, executives and business professionals exists and must be matched by building organisational management capacity in the veterinary care to promote retention and avoid further attrition of managers. The benefits and necessity of investing in developing the competency and capability of managers and building the management capacity of organisations in order to improve staff retention, service quality and organisation efficiency have been well recognised in human healthcare services.^9^, ^10^

Australia, as an example, has a workforce of 13,993 registered veterinarians^11^ and approximately 3200 registered veterinary premises.^11^ The requirement for effective leadership and management is significant to ensure the sustainability and profitability of veterinary practices and provision of high standards of care for veterinary patients.^12^ However, opportunities for veterinary professionals to develop the competencies required to be effective are extremely limited. A desktop search confirmed that only five Masters of Veterinary Science degrees are currently offered by Australian universities and none of these degrees have a clear focus on management. Although managers in veterinary care can develop their management competency by pursuing other formal tertiary education opportunities such as Masters’ of Business Administration, these degree programmes may lack a specific veterinary care context thereby limiting managers’ ability to apply this knowledge in their managerial roles.

Improved leadership and management capacity are required to address the factors challenging veterinary care services. Before strategies can be formulated, the current efforts in building leadership and management capacity and developing competency for managers and leaders in veterinary care needs to be better understood. To achieve this goal, a scoping review of the literature and all relevant information was undertaken to answer the following two questions:

What are the key challenges facing veterinary care and managing veterinary services?

What do we know about the current efforts in leadership and management capacity development and competency requirements for managers and leaders in veterinary care?

## METHODS

### Study design

A two‐step approach was adopted to search for the most up‐to‐date evidence and information to answer the above two questions.

### Step one—scoping review of research literature

The scoping review followed the six‐step process described by JBI^1^ due to the exploratory and descriptive nature of the study objectives^13^, ^14^, ^15^ and reported according to the Preferred Reporting Items for Systematic Reviews and Meta‐Analyses (PRISMA) Extension for Scoping Reviews (PRISMA‐ScR).^14^, ^15^ The scoping review included articles in the veterinary care context only. Table 1 details the key concepts and associated keywords that guided the search.

**TABLE 1:** Key concepts and keywords for the literature scoping review.

Concept one: challenges	Concept two: competency	Concept three: management	Concept four: vet services
Challenges	Competence	Manage	Veterinary
Workforce	Knowledge	Manager	Veterinary industry
Finance	Skills	Lead	Veterinary Science
Quality of care	Behaviour	Leader	Veterinarian
Demand	Competency	Leadership	Veterinary care
Stress			Veterinary science
Burnout			
Staff retention			
Mental health			

*Note*: Keywords and concept search combination—concept one and concept four, concept two and concept three and concept four

#### Article inclusion criteria

- Literature must include concept three—veterinary care services and at least concept one or two.
- Have been published in the English language since 2000.
- Are published in a peer‐reviewed journal.
- Present findings from empirical research or are classified as review articles.

Articles not meeting the above criterion were excluded from data extraction.

#### Data source and search strategy

Searches of databases were conducted in March 2023 using the combination of keywords and key concepts as detailed in Table 1. Electronic databases, including PubMed, Scopus and Web of Science, were initially accessed because this is where the majority of the articles are recorded. An additional snowball search technique of scanning reference lists to identify additional relevant articles was also applied to compliment the original search.

#### Screening

Results from keyword search were imported into the EndNote software (v.20), with duplications removed. Titles and abstracts of the articles generated by the searches were screened independently by both authors leading to the confirmation of potentially relevant articles. Once agreement was reached between both authors, full‐text review of the articles was performed by T.H. in accordance with the inclusion criteria. The final decision on the articles to be included in the data extraction was made on the basis of agreement between both authors.

#### Selection of articles and data extraction

The following information from the articles included in full‐text review was extracted and entered into an Excel spreadsheet prior to analysis: (A) surname of the first author, (B) year of publication, (C) country of origin, (D) date of publication, (E) purpose of the article, (F) study design, (G) methods used for data collection, (H) study population, (I) sectors within veterinary care, (J) sampling size, (K) response rate, (L) key challenges facing veterinary care, (M) key challenges in managing the veterinary care services, (N) key efforts in developing leadership within veterinary care and (O) leadership and competency requirements.

#### Critical appraisal

In line with the scoping review framework, we did not conduct a critical appraisal on the papers.^16^

#### Data charting and analysis

A data extraction form was developed to organise information, and this was compiled into a single spreadsheet in Microsoft Excel 2013 (Microsoft) for coding and analysis. Thematic analysis of the data was then undertaken to confirm central challenges facing veterinary care, core competency requirements for leaders and managers and the key efforts undertaken in developing these. This information was summarised in respective spreadsheets and led to the generation of results detailed later in the paper.

### Step two—a web‐based search of relevant resources

A broad search of professional institutions/groups and university postgraduate programmes that support managers working in veterinary care was conducted on 6 September 2024.

#### Search strategies

ChatGPT 3.0 was used as part of the web‐based search of relevant sources. The phrase: ‘what management programs exist to support development of leaders and managers in veterinary care’, was entered into ChatGPT 3.0. The search results are summarised in Table 4, including professional groups, university programmes and activities.

A Google search was also performed to explore details of the industry professional groups and university post‐graduate programmes, as suggested by the ChatGPT search. From these, a search was undertaken to identify which of these listed leadership and management development as part of the strategic vision and direction for their organisation as well as to search for more information about the type of training and resources offered for managers. This content was often difficult to confirm because all documentation and training materials were not available publicly in all instances and were often available to members only. These findings are summarised in Table 5 and reflect what was available publicly at the time of the search.

#### Data review, extraction and analysis

All institutions and university programmes, including relevant websites identified through the ChatGPT and Google searches, were itemised. The documentation identified from these websites was reviewed and information relevant to the training and support of managers working in veterinary care was extracted for content analysis to understand the current collected efforts in this area.

## RESULTS—SCOPING REVIEW OF LITERATURE

### Study selection

**FIGURE 1:**
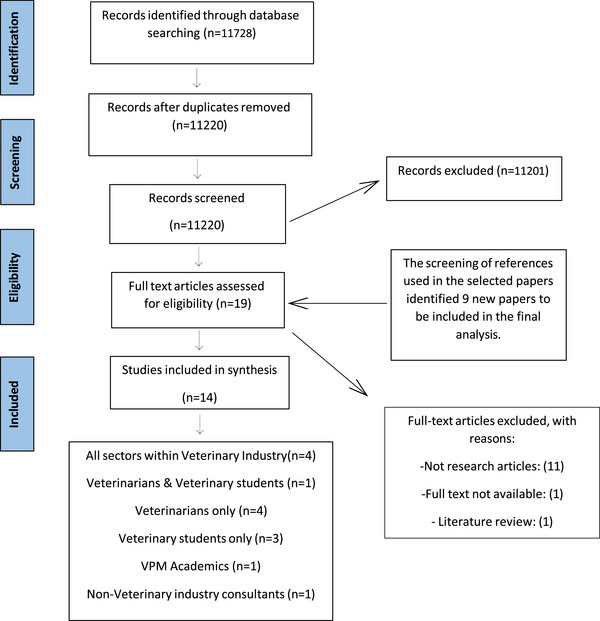
Search and review strategy (PRISMA—Preferred Reporting Items for Systematic Reviews and Meta‐Analyses—flow diagram). VPM, Veterinary Practice Management.

The PRISMA flowchart detailing the review process and search results is presented in Figure 1. In the initial search, 11,728 articles were identified within the three databases by keyword search. Title screening identified 31 potentially relevant articles. Abstract screening resulted in 19 articles being included for full‐text review. A further nine articles were excluded as a result of extended text review. Fourteen articles were progressed to the data extraction and analysis phase.

Details of the 14 articles that went through data extraction with some relevant information about the articles are summarised in Table 2.

**TABLE 2:** Details of the 14 articles included in literature search and data extraction.

Reference number	Title	Year of publication	Relevant details about the: methods for data collection, study focus and target population
1	Workplace stress, mental health and burnout of veterinarians in Australia.	2011	Postal survey of 6991 graduate veterinarians with >1 year of experience to determine the frequency of depression, anxiety, stress and burnout of veterinarians in Australia.
2	VPM: research and teaching needs as viewed by consultants and teachers.	2001	A descriptive analysis using virtual focus groups and 25 consultants and 19 VPM teachers to assess VPM education and research needs from the perspective of consultants and teachers.
3	Risk factors associated with veterinary attrition from clinical practice: a descriptive study.	2021	A descriptive study and survey of 920 Australian Veterinary Association members (practicing and retired) to understand the relationship between demographic factors and employment conditions and attrition from clinical practice.
4	Why do veterinarians leave clinical practice? A qualitative study using thematic analysis.	2020	Thematic analysis and semi‐structured interviews involving 26 veterinarians that had left clinical practice prior to retirement to understand the personal and work‐related factors that influence veterinarians to leave clinical practice.
5	Understanding veterinary leadership in practice.	2018	A phenomenological analysis and interviews with seven senior veterinary surgeons to understand what it is like to be a veterinary surgeon in an in‐practice leadership position.
6	Non‐technical competencies underlying career success as a veterinarian.	2003	A descriptive analysis including focus group discussion of 281 veterinary professionals to provide a generic definition of how success is measured in the veterinary profession, a list of non‐technical competencies (i.e., personal traits, values and abilities) that contribute to success and a set of recommendations for veterinary schools and colleges on how to select for and develop the non‐technical competencies most needed in members of the veterinary profession.
12	Template for a recommended curriculum in ‘Veterinary professional development and career success’.	2002	A descriptive study including panel and small group discussions between 38 service representatives and 250 undergraduate students to develop a detailed outline of a model curriculum that would encompass the skills, knowledge, aptitudes and attitudes deemed essential for economic success in the veterinary profession.
17	Development and evaluation of a leadership programme for veterinary students.	2001	A survey of 122 course participants to develop, deliver and evaluate a leadership programme for first‐year veterinary students.
18	National workshop on core competencies for success in the veterinary profession.	2003	A descriptive study including workshops and small group discussions between 110 delegates representing 25 veterinary colleges to present, discuss and analyse the results of the Core Competencies for Veterinary Medicine project conducted by Personnel Decisions International.
19	Skills, knowledge, aptitudes and attitudes colloquium.	2004	Small group and general session discussions between 72 representatives from all sectors of veterinary care services to discuss the findings of the NCVEI, discuss their importance for the future of the veterinary profession and develop action plans accordingly.
20	Future needs and recommendations for leadership in veterinary medicine.	2005	Structured interview questions and general session discussion between 37 representatives of all sectors of the veterinary services conducting a needs assessment of the veterinary profession, providing background for the development and implementation of programmes to enhance the leadership skills of veterinarians and veterinary students.
21	The University of California Veterinary Student Leadership Programme: comparison of a 5‐day with a 3‐day course.	2006	Qualitative data analysis of 30 veterinary students prior to commencing the first‐year science degree to describe the changes made in the curriculum, compare results of evaluations from the 5‐ and 3‐day courses and outline a method to institutionalise a similar leadership course.
22	Measurement of leadership skills development among veterinary students and veterinary professionals participating in an experiential leadership programme (the Veterinary Leadership Experience).	2019	A descriptive analysis and survey to evaluate outcomes of a experiential leadership programme among 218 participants (veterinary students and veterinarians) 1 year after completion.
23	Career paths of alumni of the Cornell Leadership Programme for veterinary students.	2008	Document analysis to understand career paths of 307 participants (veterinary students), any relationship between characteristics of students and career paths and any evidence that choice of career paths was influenced by participation in the leadership programme.

Abbreviation: VPM, Veterinary Practice Management.

### Study location and year of publication

The 14 articles presented findings of studies conducted in three countries: the USA (*n* = 10), Australia (*n* = 3) and the UK (*n* = 1). Ten of the 14 articles were published prior to 2011, demonstrating a declining trend in the publication of management competency study or studies on challenges facing the veterinary profession, in particular a significant decrease in the years 2011‒2018. It is important to acknowledge that these numbers and trends may be influenced by factors such as individual authors publishing several pieces of work over a short period of time. It is important to also acknowledge that this declining trend in articles could bias the relevance of findings; however, the continuity of findings is noted throughout articles despite date of publication.

### Sectors and target population

Of the 14 articles, three articles focused on undergraduate veterinarians and 11 articles targeted veterinarians and professionals in different management positions. Of these articles, 42% (*n* = 6) targeted study populations reflective of management experience, veterinary directors, academic deans, Veterinary Practice Management (VPM) academics and senior representatives from allied veterinary organisations and representatives. The rest were veterinarians of unspecified management levels and experience (*n* = 5, 35%).

### Methods used to identify management competency

Among the 14 articles, 10 adopted a single method, and four adopted mixed methods for data collection. The two most common methods used were surveys (*n* = 6) and semi‐structured interviews (*n* = 3). In addition, two articles used focus group discussion, two used expert panel and one performed document analysis.

### Key challenges in managing veterinary care services

Eleven out of the 14 articles discussed 13 key challenges in managing the veterinary care services. Thematic analysis confirmed that the majority of the challenges (as detailed in Table 3) could be grouped into the following three categories:

1. High rate of attrition from clinical practice.
2. High levels of depression, stress and burnout.
3. Lack of investment in leadership and management development.

**TABLE 3:** Key challenges facing veterinary care and managing the veterinary care services.

Themes	Key challenges
High rate of attrition from clinical practice.	Long hours due to high caseloads, understaffing.^1^, ^3^, ^4^
	Emergency duties in addition to regular hours.^1^, ^3^, ^4^
	Poor remuneration coupled with high education debt.^1^, ^3^, ^4^
	Negative clinical outcomes and experiences including loss of patient, surgical complications, negative client interactions/abuse led to decreased job satisfaction and career longevity.^6^, ^16^
	Negative workplace experiences, that is, poor interpersonal relationships.^9^, ^19^
High levels of depression, stress and burnout.	Personal factors such as poor mental and physical health, emotional intelligence.^1^, ^2^, ^4^, ^5^, ^6^, ^17^, ^19^, ^20^, ^24^
	Poor ability to manage work‒life balance.^5^, ^6^, ^7^, ^9^, ^15^, ^16^, ^18^, ^19^
Lack of investment in leadership and management development of veterinary undergraduates.	Lack of leadership competencies in veterinary professionals including veterinary students.^3^, ^4^, ^7^, ^11^, ^12^, ^13^, ^15^, ^18^, ^19^
	New technologies and societal expectations that require specific training in leadership and management to navigate successfully.^7^, ^13^
	Inadequate time in teaching leadership in veterinary medicine at an undergraduate level.^7^, ^12^, ^13^
Lack of investment in leadership and management development in veterinary organisations.	Low level management skill characterises veterinary profession.^13^, ^17^, ^19^
	Lack of specific training received by veterinary managers and leaders.^4^, ^5^, ^7^, ^11^, ^12^, ^13^, ^15^, ^18^, ^19^
	New technologies and societal expectations that require specific training in leadership and management to navigate successfully.^7^, ^13^

### Building leadership and management capacity in veterinary care

Among the 14 articles, none investigated or reported current efforts in building leadership and management capacity in veterinary care. Twelve articles made recommendations of what was required to overcome the recognised challenges facing veterinary care and building leadership and management capacity. These articles also confirmed that the lack of investment in leadership and management development within veterinary schools and veterinary organisations attributed to the low level of management skill that characterises the veterinary profession^2^, ^5^, ^17^ and lack of management capability development for veterinary managers and leaders.^2^, ^5^, ^16^, ^18^, ^19^, ^20^, ^24^

The articles recommended that veterinary schools/colleges should help students to develop the non‐technical skills, knowledge, attributes (SKAs) relevant to developing a successful career in leadership and management.^2^, ^3^, ^4^, ^6^, ^12^, ^19^ This included revisiting admissions processes and better understanding the undergraduate applicant pool,^2^, ^6^ and selection of students with the personality and interest profile characteristics for leadership and management.^1^ Recommendations were also made that skills relevant to leadership and management should be included in both the undergraduate and postgraduate curriculums and should form part of the accreditation requirement for veterinary schools. To achieve such a vision, the coordination of VPM education programmes at the undergraduate level, research into developing leadership and management capacity within veterinary care at the postgraduate level, and ongoing support offered to veterinary organisations at the national level are required.

### Competency requirements for managers

Eight out of the 14 articles discussed the skills or competency requirements for managers working in veterinary care. The review confirmed that no evidence‐based competency framework had been developed to guide developing managers’ competency and that no coordinated efforts had been spent in developing a competent management workforce for the veterinary care services. More than 30 skills, knowledge or competencies were extracted from the eight articles. To help grouping these 30 skills and knowledge into relevant competencies, the Management Competency Framework and Assessment tool (MCAP Framework/Tool) that was originally developed and validated in the Australian human healthcare context was used.^21^, ^25^ Table 4 details the 30 skills under the relevant management competency included in the MCAP Framework/Tool. The majority of the skills and knowledge identified in these articles were related to four of the six competencies. The remaining two competencies of knowledge of veterinary environment, organisation and enabling and managing change, did not receive much mention in the eight articles mentioned.

**TABLE 4:** Management Competency Framework and Assessment tool competencies and skills requirement for managers in veterinary clinical practice.^21^, ^24^

Competency (C)	Skills identified as being required by managers in the scoping review of literature
C1: Evidence Evidence‐informed decision making.	Decision making^5^, ^17^ Critical thinking skills^26^ Seeking answers to questions^22^ Thinking (using sound judgement, thinks innovatively)^6^
C2: Resources Operations, administration and resource management.	Human resources^2^ Strategic planning^2^ Personal and business financial management^2^ Practice/business competencies (business oriented)^6^ Practice/business management^18^
C3: Knowledge Knowledge of healthcare environment and the organisation.^a^	Understanding issue complexity^17^
C4: Communications Interpersonal, communication qualities and relationship management.	Client communications skills^5^, ^27^ Communication^2^, ^5^, ^17^, ^18^, ^22^, ^24^ Confidence in the expression of ideas^22^ Conflict management^2^, ^5^ Developing trust^24^ Honest^20^ Emotional intelligence^5^, ^28^ Interpersonal skills^5^, ^18^, ^20^ Motivation^5^, ^17^ Interpersonal competencies (build relationships)^5^, ^6^ Self‐awareness^5^, ^11^, ^17^, ^20^ Self‐confidence^20^ Self‐discipline^20^ Psychological flexibility^27^ Self‐management (acts autonomously and confidently, drives for results, demonstrates integrity, pursues development, demonstrates adaptability and resilience, communicates effectives)^6^, ^18^
C5: Leadership Leading people and organisation.	Creative^20^, ^24^ Leadership (motivates/influences/coaches and develops others)^2^, ^18^ Understanding, working in and leading organisations^20^ Visionary^20^ Teamwork^5^ team building^2^, ^17^, ^24^ Openness to learning from others^17^ Commitment beyond self‐interest^16^
C6: Change Enabling and managing change.	Lead change^20^ Resilience^28^

^a^ This item has been adapted to the veterinary care context instead of human healthcare.

### Results—step two: web‐based search of relevant resources

A search of the grey literature was conducted and is summarised in Table 5, which reports some of the professional institutions and conferences convened to support veterinary leaders and managers and their professional development. Of the 12 professional groups, university programmes and organisations identified by the grey literature search, 11 provided support to managers in developing management competencies as a part of their organisational vision or contained a management‐specific special interest group. Five institutions provided formal recognition of this training and development upon completion; however, none of these offer a postgraduate level of training, development or competency framework that guides management training design.

**TABLE 5:** Summary of the grey literature review.

Name of organisation	Website	Annual conference	Provides regular management training	Competency framework that guides management training design	Provide qualifications in management?
USA					
American Animal Hospital Association	https://www.aaha.org/	Annual conference (AAHA Con)	No	No	CVPM—certification through VHMA
Veterinary Business Management Association	https://vbma.biz/	National Meeting	Yes	No	The Business Certificate, The Business Certificate with Honours
American Veterinary Medical Association	https://www.avma.org/	Annual Veterinary Leadership Conference	Yes	No	CVPM—certification through VHMA
Penn Vet Executive Leadership Programme	https://www.vet.upenn.edu/	N/A	Yes	No	No
Veterinary Hospital Managers Association	https://www.vhma.org	Annual Meeting and Conference	Yes	No	CVPM—certification through VHMA
Veterinary Management Group	https://www.myvmg.com/	Annual conference	Yes	No	No
Veterinary Management Institute	https://www.aaha.org/resources/veterinary‐management‐institute/	Five‐month online course	Yes	No	Certificate of completion
Veterinary Practice Management Programme	https://business.purdue.edu/non‐degree/executive‐education/home.php	Six‐month online course	Yes	No	No
Australia					
Australian Veterinary Association	https://www.ava.com.au/	Annual Leadership Conference	Yes	No	Diploma of Leadership and Management
UK					
SPVS	https://spvs.org.uk/	Annual SPVS Congress	Yes	No	Institute of Leadership and Management qualification level 5
Veterinary Management Group	https://vetmg.com	Annual conference	Yes	No	Diploma in Veterinary Leadership and Management

Abbreviations: CVPM, Certificate of Veterinary Practice Management; SPVS, Society of Practising Veterinary Surgeons.

## DISCUSSION

This scoping review identified that managers working within veterinary clinical care (practice) may not have adequately acquired the necessary competencies prior to assuming management roles and that organisations may not have the capacity to support and develop managers appropriately so that they can be successful in excelling in their management roles.^5^, ^6^

The importance of effective management capability to develop solutions and address factors contributing to the high rates of attrition from clinical practice has been well documented.^3^, ^4^, ^17^ Although there have been efforts made towards developing leadership and management capability in veterinary care, the scoping review found that no coordinated efforts have been made to identify the core management competencies required to develop such capability or to guide the coordinated implementation of these at an undergraduate and postgraduate level. In addition, although some management related skills, knowledge and competencies were mentioned in the identified articles,^1^, ^17^, ^27^ they are far from adequate in representing the core competency requirements of managers based on evidence from human healthcare services.^28^ In particular, the lack of reinforcement of competencies of understanding the veterinary care and organisational context, and leading and managing changes, are problematic as they are integral for managers to work in rapidly evolving veterinary organisations. The capability of veterinary managers in leading and managing transitions through change is critical, especially given the rapidity with which inevitable challenges face the profession.

Veterinary care needs a comprehensive management competency framework that can guide leadership and management development within the veterinary context. The growing interest in management competency studies in both the human health and non‐health industries in the past 10 years, the investment in management competency development and experience learned from the human healthcare services^29^ have proven that it is time for investment in developing a competent management workforce for veterinary care services in a more systematic manner. Evidence from human healthcare services indicates that competent managers have a positive effect on quality patient care,^29^, ^30^ and staff retention,^31^, ^32^, ^33^ and that improved managers’ competency can lead to improved service outcomes.^33^, ^34^ Continued professional development opportunities are essential for individual development, staff retention and health system capacity building.^35^ Veterinary care services and organisations need the opportunities and guidance to develop their managers in a more systematic manner, leading to better support to clinical staff and quality care provision. The increasing development of professional institutions and convening of annual leadership and management conferences globally demonstrate the necessity of developing better management capacity within the veterinary care services, as evidenced by the grey literature search. However, in the absence of a comprehensive management competency framework to guide developing management capacity, programmes and resources are allocated more on an ad hoc basis. A competency framework for managers needs to be established to guide the design of such professional development, training and education in a more systematic manner, to support and develop the management workforce within veterinary care. This calls for a more coordinated and national approach that strengthens collaboration between government/funding bodies, veterinary schools, veterinary training organisations and veterinary organisations, recognising the important roles that managers play and guiding investment in building management workforce capability.

The lack of identified articles on management capability development in veterinary care indicates that more investigation into understanding the competency development needs of veterinary managers is necessary to provide evidence to guide formulating strategies for veterinary management workforce development. Such evidence can guide the design of formal and more systematic training, such as postgraduate programmes in management for veterinary professionals, that develop their management competencies required to be effective managers within the veterinary services. However, developing formal management programme tailored for veterinary managers is a goal that takes time to achieve. Hence, the authors argue that the necessity of reviewing the current undergraduate curriculums globally and inclusion of non‐clinical component such as in the areas of leadership and management for current formal educational programmes to veterinary professionals is a more feasible immediate and medium‐term goal and highlights an important area for future research and analysis.^27^

The declining rate of articles into management competency studies in the veterinary services also highlights the lack of acknowledgement and priority in the criticality of developing management capacity to support sustainable practices and careers in the veterinary services. Of the nine articles in this area, only five were undertaken after 2010, including the two after 2019. When the challenges facing the veterinary services have been at their greatest, the declining trend is at odds with services requirement. Investment in researching in identifying the core management competencies is urgently required to provide solutions to the new and emerging strategic challenges facing the veterinary services both now and into the future. Building management research capacity in the veterinary services is also important to encourage evidence‐based practices and decision making to improve quality of veterinary care and the effectiveness and efficiency of veterinary service provision.

In acknowledging the limitations in using an artificial intelligence engine (ChatGPT) in the methods of this study, it is important to acknowledge that the Google search will be biased to the geographical location where the search is undertaken. This is a limitation of this method of grey literature search and could be mitigated by future searches specifically undertaken for geographical regions to reflect more complete search results of the support available to leaders and managers globally. It is important to also note that using this method of grey literature search only identified the resources that were publicly searchable at the time of the review and therefore represents only some of the available support to leaders and managers; it is not an extensive or complete review. This is an acknowledged limitation of this method of grey literature search.

Identifying and developing management competencies for the veterinary services is necessary to find sustainable solutions to the challenges that plague the services. This review confirmed the limited efforts to date (in terms of published articles) in developing management capability and the lack of understanding of management requirements for managers working in the veterinary services and argued the necessity of developing a management competency development framework that guides the development management capability. Such a framework can also be utilised to guide position recruitment, career development and succession planning for managers working in veterinary services as well as the development of a competency‐based curricular at both the undergraduate and postgraduate levels. This scoping review confirmed the requirement for effective leadership and management of the veterinary workforce and that a validated management competency framework to guide the development of this management capacity is required in order to ensure sustainable and profitable veterinary practices, career pathways for veterinary professionals, standards of care for veterinary patients and support for the global community.

## AUTHOR CONTRIBUTIONS

Taleta Hompas and Zhanming Liang were responsible for the conception and design of the study. Taleta Hompas was responsible for the initial drafting of the manuscript. Zhanming Liang revised, edited and proofread the complete manuscript. Both Taleta Hompas and Zhanming Liang approved the final version before submission.

## CONFLICTS OF INTEREST

The authors declare they have no conflicts of interest.

## ETHICS STATEMENT

The authors confirm that the ethical policies of the journal, as noted on the journal's author guidelines page, have been adhered to. No ethical approval was required because this was a review article with no original research data.

## Data Availability

The datasets used and/or analysed during the current study are available from the corresponding author upon reasonable request.
